# Supplementary material for: Risk factors for postpartum haemorrhage in the Northern Province of Rwanda: A case control study
Source: PLoS One. 2022 Feb 15;17(2):e0263731. doi: 10.1371/journal.pone.0263731 (PMC8846539; doi:10.1371/journal.pone.0263731)
Supplement: S1 Checklist — (PDF) [file pone.0263731.s002.pdf]

Supplementary information: STROBE checklist

Manuscript title

"Investigating and modelling risk factors for primary postpartum haemorrhage among childbearing Women in the Northern Province of Rwanda: A case control study".

Authors: Oliva Bazirete; Manassé Nzayirambaho, Aline Umubyeyi, Innocent Karangwa, Marilyn Evans

	Item No	Recommendation	Page No
**Title and abstract**	1	(*a*) Indicate the study's design with a commonly used term in the title or the abstract	1-2
		(*b*) Provide in the abstract an informative and balanced summary of what was done and what was found	2
**Introduction**			
Background/rationale	2	Explain the scientific background and rationale for the investigation being reported	3-5
Objectives	3	State specific objectives, including any prespecified hypotheses	5
**Methods**			
Study design	4	Present key elements of study design early in the paper	5
Setting	5	Describe the setting, locations, and relevant dates, including periods of recruitment, exposure, follow-up, and data collection	5
Participants	6	(*a*) Give the eligibility criteria, and the sources and methods of case ascertainment and control selection. Give the rationale for the choice of cases and controls	6
		(*b*) For matched studies, give matching criteria and the number of controls per case	Unmatched study
Variables	7	Clearly define all outcomes, exposures, predictors, potential confounders, and effect modifiers. Give diagnostic criteria, if applicable	8
Data sources/ measurement	8*	For each variable of interest, give sources of data and details of methods of assessment (measurement). Describe comparability of assessment methods if there is more than one group	7
Bias	9	Describe any efforts to address potential sources of bias	6
Study size	10	Explain how the study size was arrived at	6-7
Quantitative variables	11	Explain how quantitative variables were handled in the analyses. If applicable, describe which groupings were chosen and why	9-10
Statistical methods	12	(*a*) Describe all statistical methods, including those used to control for confounding	9-10
		(*b*) Describe any methods used to examine subgroups and interactions	9-10
		(*c*) Explain how missing data were addressed	9
		(*d*) If applicable, explain how matching of cases and controls was addressed	N/A
		(*e*) Describe any sensitivity analyses	N/A

	Item No	Recommendation	Page No
**Results**			
Participants	13*	(a) Report numbers of individuals at each stage of study—eg numbers potentially eligible, examined for eligibility, confirmed eligible, included in the study, completing follow-up, and analysed	7<br>See Figure
		(b) Give reasons for non-participation at each stage	7
		(c) Consider use of a flow diagram	7
Descriptive data	14*	(a) Give characteristics of study participants (eg demographic, clinical, social) and information on exposures and potential confounders	10
		(b) Indicate number of participants with missing data for each variable of interest	11
Outcome data	15*	Report numbers in each exposure category, or summary measures of exposure	12, table 2
Main results	16	(*a*) Give unadjusted estimates and, if applicable, confounder-adjusted estimates and their precision (eg, 95% confidence interval). Make clear which confounders were adjusted for and why they were included	15 table 3
		(*b*) Report category boundaries when continuous variables were categorized	13, table 2
		(*c*) If relevant, consider translating estimates of relative risk into absolute risk for a meaningful time period	15 table 3
Other analyses	17	Report other analyses done—eg analyses of subgroups and interactions, and sensitivity analyses	N/A
**Discussion**			
Key results	18	Summarise key results with reference to study objectives	15-19
Limitations	19	Discuss limitations of the study, taking into account sources of potential bias or imprecision. Discuss both direction and magnitude of any potential bias	19
Interpretation	20	Give a cautious overall interpretation of results considering objectives, limitations, multiplicity of analyses, results from similar studies, and other relevant evidence	20
Generalisability	21	Discuss the generalisability (external validity) of the study results	20
**Other information**			
Funding	22	Give the source of funding and the role of the funders for the present study and, if applicable, for the original study on which the present article is based	21

Note: The above checklist was based on available evidence[1, 2].

References

1. von Elm E, Altman D, Egger M, Pocock S, Gtzsche P, Vandenbroucke J, et al. The Strengthening the Reporting of Observational Studies in Epidemiology (STROBE) Statement: Guidelines for reporting observational studies. International journal of surgery (London, England). 2014;12:1495-9.
2. Vandenbroucke JP, Elm Ev, Altman DG, Gøtzsche PC, Mulrow CD, Pocock SJ, et al. Strengthening the Reporting of Observational Studies in Epidemiology (STROBE): Explanation and Elaboration. PLoSMEDICINE. 2007.
