# Supplementary material for: Risk factors for postpartum haemorrhage in the Northern Province of Rwanda: A case control study
Source: PLoS One. 2022 Feb 15;17(2):e0263731. doi: 10.1371/journal.pone.0263731 (PMC8846539; doi:10.1371/journal.pone.0263731)
Supplement: Attachment — Submitted filename: Response to reviewers.docx [file pone.0263731.s003.docx]

**Response to reviewers:**

**Risk factors for** **postpartum haemorrhage in the Northern Province of Rwanda: a case control study**

| **No** | **Reviewer’s comments** | **Changes/ Response made** | **Response inscription in the manuscript with highlights** |
| --- | --- | --- | --- |
| **Journal requirements** | | | |
| **1.** | Please ensure that your manuscript meets PLOS ONE's style requirements, including those for file naming | Files naming changed, and PLOS One style requirements followed in the whole manuscript (Format of headings, line numbering) | Title page 1, L 1-2  The entire manuscript |
| **2.** | Please ensure that you have an ORCID iD and that it is validated in Editorial Manager. | My ORCID Id: [https://orcid.org/0000-0002-8476-3448](https://orcid.org/0000-0002-8476-3448?lang=en) | ORCID Id validated in editorial manager  Title page 1, L 12 |
| **3.** | Your ethics statement should only appear in the Methods section of your manuscript. If your ethics statement is written in any section besides the Methods, please delete it from any other section. | Ethics statement deleted from abstract and from declarations and only placed in Methods section | Page 5, L134-139 |
| **4.** | Please include captions for your Supporting Information files at the end of your manuscript, and update any in-text citations to match accordingly | Hyperlinks created for fig 1, table1, 2and 3  Captions for supporting information files (S1 dataset) updated and included at the end of the manuscript | L190,295,315,326 and 339  Page 22, L 525 |
| **5.** | Please review your reference list to ensure that it is complete and correct. | List of reference reviewed and updated | Pages 23-31, L541-770 |
| **6** | We noticed you have some minor occurrence of overlapping text with the following previous publication(s), which needs to be addressed: <https://bmcpregnancychildbirth.biomedcentral.com/articles/10.1186/s12884-016-1217-0>  Address this in the Strengths & limitations section. | Overlap in the text addressed.  The section of strengths & limitation has been revised to avoid text overlap and Nyflot [2017] is cited | Page 21-22, L495-509 |
| **Reviewer 1 comments** | | | |
| **1** | Under Methods, at the end of the second last paragraph clearly state that a P value of 0.05 was taken as statistical significant | Level of significance clearly defined | Page 11,L283-284 |
| **2** | I note that you state that p values 0.077 are statistically significant, which is not correct. Round off all p values to 2 decimal places except those which are 0.0001.  Also report your RR in 2 decimal places as well | Risk factors with a P greater than p=0.05 were removed ( intrauterine fetal death and premature rupture of membranes of P: 0.08)  All p values Rounded off to 2 decimal places except those which are <0.0001  RR neatly reported in 2 decimal places both in table 3 and in texT | Correction made in abstract, findings and conclusion  L 314-357  L336-357 |
| **Reviewer 2 comments** | | | |
| **1** | The title is rather lengthy, reduction is suggested for example into “Risk factors for postpartum haemorrhage in Northern Province of Rwanda: a case control study” | New title is shortened:  Risk factors for postpartum haemorrhage in Northern Province of Rwanda: a case control study” | Title page, L1-2 |
| **2** | **Background**  Typo page 3, 2nd paragraph line 6: helps has to be help | Typo corrected | L 69 |
| **3** | **Background**: Typo page 3, 2nd paragraph last sentence, “in Come countries”, has to be income countries. | Typo corrected | L77 |
| **4** | **Background:** The authors are invited to elaborate on their adaptation of the PPH definition for the low and middle income countries. Not only in relation to the WHO definitions but also in relation to WHO near miss definitions. | Change to the background section was made to adapt the definition of PPH to maternal near- miss “a woman who nearly died but survived a complication that occurred during pregnancy, childbirth or within 42 days of termination of pregnancy” [WHO, 2011] | L77-81 |
| **5** | **Methods:**   The authors are invited to inform whether blood transfusions were scored within all the variables and also if the number of blood transfusions were scored. | Clarifications about blood transfusion provided | L 177-178  L 298-299 |
| **6** | **Discussion**  The authors are invited to elaborate on this subject**,** adequate preparation and plan for early intervention to prevent PPH (Fawcus 2019 presenting practical approached to managing PPH with limited resources) | Suggestion with reference added to stress on adequate preparation and plan for early intervention to prevent PPH | L407-413 |
| **7** | Page 18-19 the authors advise regular antenatal check-ups to detect risk factors for PPH and refer to reference 82: Can the authors present their own advice on how regular these check-ups ideally should be, or should be strived for. | The implementation of the new WHO ANC guidelines is suggested to ensure regular checkups during pregnancy | L 436-439 |
| **8** | Page 20, the authors mention introduction of active management at the third stage of labour.  the authors are in line with prior comments to add practical recommendations of their own or other investigators working in the same setting. Not only medical intervention with AMSTL with oxytocin but also possible mechanical intervention with bimanual uterine compression/ balloon/condom tamponade. | Other mechanical interventions to manage PPH are added and reference provided | L 508-514 |
